# Tuberculosis - The master of disguise: A case of disseminated tuberculosis masquerading as metastatic cancer

**DOI:** 10.5578/tt.202402915

**Published:** 2024-06-12

**Authors:** Ezgi Aysu ŞAHİN MAVİ, Muhammed Çağrı AKDEMİR, Ali Eren AKIN, Oğuz Abdullah UYAROĞLU, Deniz KÖKSAL

**Affiliations:** 1 Department of Internal Medicine, Hacettepe University Faculty of Medicine, Ankara, Türkiye; 2 Department of Pathology, Hacettepe University Faculty of Medicine, Ankara, Türkiye; 3 Department of Chest Diseases, Hacettepe University Faculty of Medicine, Ankara, Türkiye

## Abstract

**ABSTRACT**

**
Tuberculosis - The master of disguise: A case of disseminated tuberculosis masquerading as metastatic cancer
**

*
Tuberculosis (TB) is an airborne infectious disease caused by Mycobacterium tuberculosis (MTB). Although it typically affects the lungs (pulmonary TB), one-fifth of TB cases present as extrapulmonary TB. The diagnosis of extrapul- monary TB is often overlooked due to its atypical clinical and radiological manifestations. Differentiating TB from neoplastic conditions poses signifi- cant challenges. A 33-year-old female patient was admitted to the emergency clinic with shortness of breath, cough, and abdominal pain. Postero-anterior chest X-ray revealed massive pleural effusion leading to mediastinal shift. With a preliminary diagnosis of malignant pleural effusion, a pleural catheter was inserted, and the patient was referred for a positron emission tomogra- phy (PET/CT) to assess the primary site and the optimal location for a biopsy. The PET/CT revealed asymmetric soft tissue thickening on the left side of the nasopharynx, and increased fluorodeoxyglucose (FDG) uptake in the left cervical lymph nodes raised suspicion regarding primary nasopharyngeal cancer. Additionally, there was an increased FDG uptake observed in the mass lesion located in the right upper lobe, mediastinal lymph nodes, pleural surfaces in the left hemithorax, perihepatic areas, and peritoneum, indicating diffuse metastatic disease. Tuberculosis diagnosis was confirmed through biopsies demonstrating granulomatous inflammation in the lung and naso- pharynx, along with culturing MTB from pleural effusion. Positron emission tomography played a crucial role in identifying sites of TB involvement. Despite its rarity, healthcare professionals should consider nasopharyngeal TB as a potential diagnosis when evaluating nasopharyngeal masses.
*

**Key words:** *Tuberculosis; extrapulmonary tuberculosis; nasopharyngeal tuberculosis; nasopharyngeal carcinoma
*

**ÖZ**

**
Tüberküloz - Kılık değiştirme ustası: Metastatik kanseri taklit eden bir dissemine tüberküloz vaka sunumu
**

*
Tüberküloz (TB), Mycobacterium tuberculosis’in (MTB) neden olduğu, hava yoluyla bulaşan bulaşıcı bir hastalıktır. Tipik olarak akci- ğerleri (akciğer TB) etkilemesine rağmen, TB vakalarının beşte biri akciğer dışı TB olarak ortaya çıkar. Atipik klinik ve radyolojik bul- gular nedeniyle ekstrapulmoner TB tanısı sıklıkla gözden kaçırılmaktadır. Tüberkülozu neoplastik durumlardan ayırmak önemli zor- luklar doğurur. Bu olgu sunumunda nefes darlığı, öksürük ve karın ağrısı şikayetiyle acil servise başvuran 33 yaşında bir kadın hasta ele alınmıştır. Postero-anterior akciğer grafisinde mediastinal kaymaya yol açan masif plevral efüzyon görülen hastaya malign plevral efüzyon ön tanısıyla plevral kateter yerleştirildi ve hasta, primer bölgenin ve biyopsi için en uygun yerin belirlenmesi amacıyla pozit- ron emisyon tomografisine (PET/BT) yönlendirildi. Plevral emisyon tomografide nazofarenksin sol tarafında asimetrik yumuşak doku kalınlaşmasının görülmesi ve sol servikal lenf nodlarında florodeoksiglukoz (FDG) tutulumunun artması primer nazofarenks kanseri şüphesini artırdı. Ayrıca sağ üst lobda yer alan kitle lezyonunda, mediastinal lenf nodlarında, sol hemitorakstaki plevral yüzeylerde, perihepatik alanlarda ve peritonda yaygın metastatik hastalığa işaret edecek şekilde artmış FDG tutulumu tespit edildi. Tüberküloz tanısı akciğer ve nazofarenkste granülomatöz inflamasyonu gösteren biyopsilerle ve plevral efüzyondan MTB kültürüyle doğrulandı. Plevral emisyon tomografi, TB tutulum bölgelerinin belirlenmesinde çok önemli bir rol oynadı. Nadir görülmesine rağmen, sağlık profesyonelleri nazofaringeal kitleleri değerlendirirken nazofaringeal TB’yi potansiyel bir tanı olarak düşünmelidir.
*

**Anahtar kelimeler:** *Tüberküloz; ekstrapulmoner tüberküloz; nazofaringeal tüberküloz; nazofaringeal karsinom
*

## INTRODUCTION

Tuberculosis (TB) is an airborne infectious disease caused by *Mycobacterium tuberculosis* (MTB). It is the leading infectious cause of death in adults worldwide. Nearly two billion people (about one- quarter of the world population) are estimated to be infected with MTB. More than 10 million people continue to fall ill with TB every year (1). Although TB typically affects the lungs (pulmonary TB), one-fifth of TB cases present as extrapulmonary TB, among which the most common sites include the lymph nodes, pleura, peritoneum, bone and joints, genitourinary system, abdomen, and central nervous system (2). Head and neck TB constitutes 10% of TB cases and can affect various organs in the head and neck region, including lymph nodes, larynx, middle ear, oral cavity, and pharynx (3). Although nasopharyngeal TB has been identified in less than 1% of TB cases, recent literature suggests an increased awareness or incidence of this condition. Furthermore, the most frequent presenting symptom is cervical lymphadenopathy, which can mimic nasopharyngeal carcinoma, a more prevalent and serious disease (4).

The diagnosis of extrapulmonary TB is often overlooked due to its atypical clinical and radiological manifestations. Differentiating TB from neoplastic conditions poses significant challenges. The confusion between malignancies and TB can result in misdiagnoses, delayed treatment initiation, and unnecessary diagnostic procedures (5). In this report, we present a rare case of disseminated TB involving the nasopharynx, lung, pleura, peritoneum, and lymph nodes, who was initially evaluated with a diagnosis of metastatic carcinoma of the lung and nasopharynx.

## CASE REPORT

A 33-year-old female patient was admitted to the emergency clinic with shortness of breath, cough, and abdominal pain. Over the past three months, she had a substantial weight loss of 22 kilograms. In her medical history, she was diagnosed with pseudotumor cerebri two years ago and has been receiving acetazolamide twice a day since then. She was a non-smoker and was employed as a housewife. On physical examination, decreased breath sounds and dullness to percussion were detected on the left hemithorax. Postero-anterior (PA) chest X-ray revealed massive pleural effusion in the left hemithorax, leading to mediastinal shift to the right. A computed tomography (CT) of the thorax showed the presence of a loculated pleural effusion within the left hemithorax and a mass lesion (35x22 mm) accompanied by centroacinar opacities in the right upper lobe (Figure 1). Meanwhile, an abdominal CT revealed minimal pelvic fluid accumulation and thickening of the peritoneum, interpreted as peritoneal carcinomatosis originating from a non-gynecological malignancy. Complete blood count and serum biochemical analysis were within normal limits. C-reactive protein level was mildly elevated (3.51 mg/dL), and erythrocyte sedimentation rate was 84 mm/hr. Since the patient was dyspneic due to massive pleural effusion in the left hemithorax, a therapeutic thoracenthesis (1.250 mL) was performed. As the patient remained dyspneic the following day, a pleural catheter was inserted. With a preliminary diagnosis of malignant pleural effusion, the patient was referred for a positron emission tomography (PET/CT) to assess the primary site and the optimal location for a biopsy. The PET/CT revealed asymmetric

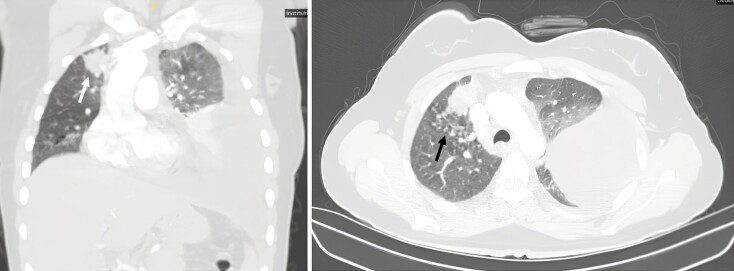

**Figure 1.** Computed tomography of thorax demonstrating the presence of a loculated pleural effusion within the left hemithorax **(A)**

and a mass lesion (35x22 mm) (**A**, white arrow) accompanied by centroacinar opacities (**B**, black arrow) in the right upper lobe.

soft tissue thickening on the left side of the nasopharynx, and increased fluorodeoxyglucose (FDG) uptake in the left cervical lymph nodes raised suspicion regarding primary nasopharyngeal cancer. Additionally, there was an increased FDG uptake observed in the mass lesion located in the right upper lobe, mediastinal lymph nodes, pleural surfaces in the left hemithorax, perihepatic areas, and peritoneum, indicating diffuse metastatic disease (Figure 2).

The pleural fluid was an exudate, with a protein level of 5.6 g/dL, albumin level of 3.22 g/dL, LDH level of 268 U/L, glucose level of 84 mg/L, and adenosine deaminase (ADA) level of 40.7 U/L. Pleural fluid cytology revealed the presence of mesothelial cells, macrophages, and lymphocytes. Aerobic pleural fluid cultures yielded negative results. A Quantiferon test was positive. A tru-cut biopsy taken from mass lesion in the right upper lobe and a punch biopsy taken from the left lateral pharyngeal recess (fossa of Rosenmüller) was both reported as granulomatous inflammation and necrosis (Figure 3). Based on the two confirmatory biopsies and the elevated ADA level in the pleural fluid, the patient was diagnosed with disseminated tuberculosis involving the nasopharynx, lung, pleura, peritoneum, and lymph nodes. Treatment was initiated with standard anti- tuberculosis therapy including isoniazid 300 mg/day, rifampicin 600 mg/day, pyrazinamide 2000 mg/day, and ethambutol 1500 mg/day. After two weeks of therapy, the patient’s complaints showed improvement, and a significant resolution of the radiological findings was observed in the PA chest X-ray. *Mycobacterium tuberculosis* was cultured from

the pleural fluid, which was susceptible to all first- line anti-tuberculosis drugs.

## DISCUSSION

Herein, we report a rare case of disseminated TB involving the nasopharynx, lung, pleura, peritoneum, and lymph nodes in an immunocompetent patient. The patient was initially evaluated with a diagnosis of metastatic carcinoma of the lung and nasopharynx. Positron emission tomography played an important role in the diagnosis and determination of the extent of the disease.

Tuberculosis is notorious for its capacity to mimic malignancies, often leading to delayed diagnosis due to shared clinical symptoms, similarities in radiological features, and challenges in bacilli isolation (5). On the other hand, patients with lung cancer are sometimes misdiagnosed as having pulmonary TB, especially in countries with a high burden of TB (6). Positron emission tomography is a metabolic imaging technique used for diagnosing and staging various malignancies. However, it cannot reliably differentiate active TB from malignant lesions since both conditions exhibit increased FDG uptakes. Additionally, PET/CT has emerged as a promising imaging modality in the field of infection and inflammation, especially for determining the extension and severity of the disease (7,8). In case of TB, it proves particularly useful in detecting the disease in previously unknown sites, mostly extrapulmonary, and facilitates the identification of the most appropriate site of biopsy (9). The integration of imaging findings with microbiological, clinical, and laboratory data enhances the accuracy of TB

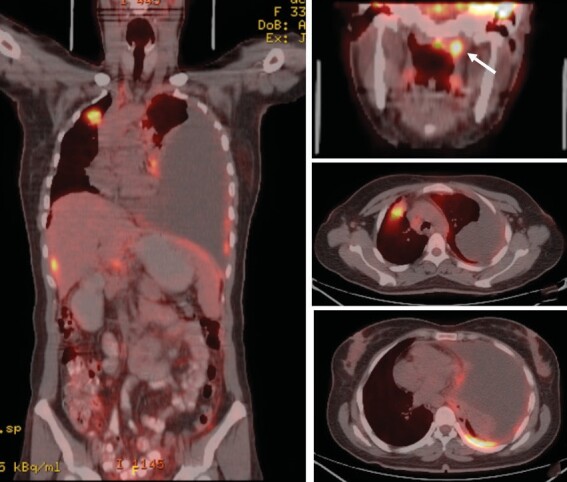

**Figure 2.** An increased FDG uptake in the mass lesion located in the right upper lobe **(A, C)**, pleural surfaces in the left hemithorax

**(A, D)**, perihepatic areas, and peritoneum **(A)**, and asymmetric soft tissue thickening on the left side of the nasopharynx **(B)**.

diagnosis and AIDS in developing an effective treatment plan.

The presented patient was hospitalized for the evaluation of a massive pleural effusion, initially thought to be of malignant etiology due to a metastatic carcinoma. The patient was referred for a PET/CT scan to assess the primary site and determine the optimal location for a biopsy. Asymmetric soft tissue thickening on the left side of the nasopharynx, along with increased FDG uptake in the left cervical lymph nodes raised suspicion regarding primary nasopharyngeal carcinoma. Additionally, increased FDG uptake was observed in the mass lesion located in the right upper lobe, mediastinal lymph nodes, pleural surfaces in the left hemithorax, perihepatic areas, and peritoneum, indicating diffuse metastatic

disease. Although TB was considered in the differential diagnosis due to a moderately elevated ADA level in the pleural effusion, biopsies from the right upper lobe pulmonary lesion and nasopharyngeal lesion were planned. Both biopsies were compatible with granulomatous inflammation and necrosis, leading to a diagnosis of disseminated TB involving the nasopharynx, lung, pleura, peritoneum, and lymph nodes. The demonstration of characteristic caseating granulomas on a tissue section in the appropriate clinical and epidemiologic circumstances strongly supports a diagnosis of active TB, but it is not pathognomonic; culture is required to establish a laboratory diagnosis and to perform drug susceptibility testing (10). While TB cultures from both biopsies were negative, MTB was cultured from pleural

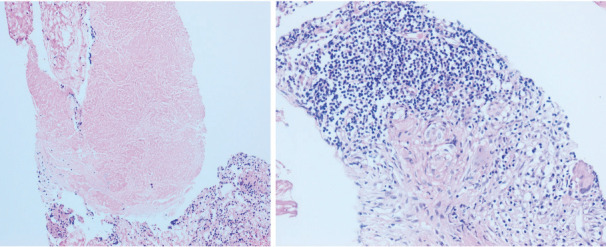

**Figure 3.** Core needle biopsy of pulmonary mass lesion showing granulomatous inflammation with lymphocytes and multinucleated giant cell (Hematoxylin & Eosin, 200x) **(A)** and necrosis (Hematoxylin & Eosin, 100x) **(B)**.

effusion, confirming the diagnosis of TB using a gold standard method. Since the bacillus was susceptible to all first line TB drugs, the patient showed significant improvement after two weeks of therapy.

Nasopharyngeal TB is a rare clinical entity that presents a diagnostic challenge due to its striking similarity to nasopharyngeal carcinoma (11). It is typically caused by the spread of bacilli through the blood, lymphatic system, or respiratory transmission

(12). Importantly, it can develop in a seemingly healthy patient without any underlying disease or immunosuppression. Nasopharyngeal tuberculosis exhibits a spectrum of clinical presentations, ranging from asymptomatic cases to symptoms that can manifest as either systemic or nasopharyngeal. The most prevalent symptom is the enlargement of cervical lymph nodes. Although nasopharyngeal TB is considered an uncommon condition, increased awareness of the disease, improvement in imaging, and diagnostic techniques have led to more frequent reports in the literature (4). In the presented case, PET/CT imaging facilitated the diagnosis of nasopharyngeal TB, which was initially misdiagnosed as nasopharyngeal carcinoma.

## CONCLUSION

In conclusion, we presented a rare case of disseminated TB masquerading as metastatic cancer. Positron emission tomography imaging clearly identified sites of TB involvement, including the nasopharynx, lung, pleura, peritoneum, and lymph nodes. The diagnosis of TB was confirmed through

biopsies demonstrating granulomatous inflammation in the lung and nasopharynx, along with culturing MTB from pleural effusion. Despite its rarity, healthcare professionals should consider nasopharyngeal TB as a potential diagnosis when evaluating nasopharyngeal masses.

## CONFLICT of INTEREST

The authors have no conflict of interest to declare.

## AUTHORSHIP CONTRIBUTIONS

Concept/Design: EASM, OAU, DK Analysis/Interpretation: EASM, OAU, DK Data acqusition: All of authors

Writing: All of authors

Clinical Revision: All of authors Final Approval: All of authors

## REFERENCES

1. *
  Global tuberculosis report 2023. Geneva: World Health Organization; 2023. Licence: CC BY-NC-SA 3.0 IGO.
  *
2. *
  Gambhir S, Ravina M, Rangan K, Dixit M, Barai S, Bomanji J. International atomic energy agency extra-pulmonary TB consortium. Imaging in extrapulmonary tuberculosis. Int J Infect Dis 2017; 56: 237-47. https://doi.org/10.1016/j.ijid.2016.11.003*
3. *
  Menon K, Bem C, Gouldesbrough D, Strachan DR. A clin- ical review of 128 cases of head and neck tuberculosis presenting over a 10-year period in Bradford, UK. J Laryngol Otol 2007; 121: 362-8. https://doi.org/10.1017/S0022215106002507*
4. *
  Srivanitchapoom C, Sittitrai P. Nasopharyngeal tuberculo- sis: Epidemiology, mechanism of infection, clinical manifes- tations, and management. Int J Otolaryngol 2016; 2016: 4817429. https://doi.org/10.1155/2016/4817429*
5. *
  Prytz S, Hansen JL. A follow-up examination of patients with pulmonary tuberculosis resected on suspicion of tumour. Scand J Respir Dis 1976; 57(5): 239-46.
  *
6. *
  Bhatt M, Kant S, Bhaskar R. Pulmonary tuberculosis as differential diagnosis of lung cancer. South Asian J Cancer 2012; 1(1): 36-42. https://doi.org/10.4103/2278-330X.96507330X.96507*
7. *
  Soussan M, Brillet PY, Mekinian A, Khafagy A, Nicolas P, Vessieres A, et al. Patterns of pulmonary tuberculosis on FDG-PET/CT. Eur J Radiol 2012; 81(10): 2872-6. https://doi.org/10.1016/j.ejrad.2011.09.002*
8. *
  Vorster M, Sathekge MM, Bomanji J. Advances in imaging of tuberculosis: The role of 18F-FDG PET and PET/CT. Curr Opin Pulm Med 2014; 20(3): 287-93. https://doi.org/10.1097/MCP.0000000000000043*
9. *
  Yu WY, Lu PX, Assadi M, Huang XL, Skrahin A, Rosenthal A, et al. Updates on 18F-FDG-PET/CT as a clinical tool for tuberculosis evaluation and therapeutic monitoring. Quant Imaging Med Surg 2019; 9(6): 1132-46. https://doi.org/10.21037/qims.2019.05.24*
10. *
  WHO consolidated guidelines on tuberculosis. Module 3: Diagnosis. Tests for tuberculosis infection. Geneva: World Health Organization; 2022. Licence: CC BY-NC-SA 3.0 IGO.
  *
11. *
  Oon A, Razuan NA, Kuppan G, Mahadzir M. Nasopharyngeal tuberculosis mimicking nasopharyngeal carcinoma: A case series. Indian J Otolaryngol Head Neck Surg 2022; 74(Suppl 2): 1612-4. https://doi.org/10.1007/s12070-021-02770-y*
12. *
  Nakao Y, Shibata R, Murohara T, Tanigawa T. Primary naso- pharyngeal tuberculosis: A case report. BMC Infect Dis 2016; 16:121. https://doi.org/10.1186/s12879-016-1449-7*

